# Development of Relaxin-3 Agonists and Antagonists Based on Grafted Disulfide-Stabilized Scaffolds

**DOI:** 10.3389/fchem.2020.00087

**Published:** 2020-02-18

**Authors:** Han Siean Lee, Michael Postan, Angela Song, Richard J. Clark, Ross A. D. Bathgate, Linda M. Haugaard-Kedström, K. Johan Rosengren

**Affiliations:** ^1^Faculty of Medicine, School of Biomedical Sciences, The University of Queensland, Brisbane, QLD, Australia; ^2^Florey Department of Neuroscience and Mental Health, Florey Institute of Neuroscience and Mental Health, The University of Melbourne, Melbourne, VIC, Australia; ^3^Department of Biochemistry and Molecular Biology, The University of Melbourne, Melbourne, VIC, Australia

**Keywords:** relaxin-3, RXFP3, grafting, apamin, VhTI, disulfide-scaffold

## Abstract

Relaxin-3 is a neuropeptide with important roles in metabolism, arousal, learning and memory. Its cognate receptor is the relaxin family peptide-3 (RXFP3) receptor. Relaxin-3 agonist and antagonist analogs have been shown to be able to modulate food intake in rodent models. The relaxin-3 B-chain is sufficient for receptor interactions, however, in the absence of a structural support, linear relaxin-3 B-chain analogs are rapidly degraded and thus unsuitable as drug leads. In this study, two different disulfide-stabilized scaffolds were used for grafting of important relaxin-3 B-chain residues to improve structure and stability. The use of both *Veronica hederifolia* Trypsin inhibitor (VhTI) and apamin grafting resulted in agonist and antagonist analogs with improved helicity. VhTI grafted peptides showed poor binding and low potency at RXFP3, on the other hand, apamin variants retained significant activity. These variants also showed improved half-life in serum from ~5 min to >6 h, and thus are promising RXFP3 specific pharmacological tools and drug leads for neuropharmacological diseases.

## Introduction

Relaxin-3 is a neuropeptide that belongs to the insulin/relaxin superfamily of peptide hormones (Bathgate et al., 2002). Its endogenous receptor, the relaxin family peptide-3 (RXFP3) receptor, is a G_i/o_-protein coupled receptor (GPCR) (Liu et al., 2003). The relaxin-3/RXFP3 network, which is primarily expressed in the nucleus incertus (NI) and projects broadly throughout the brain, is known to modulate several physiological processes (Ma et al., 2017). NI neurons expressing relaxin-3 are highly responsive to stress signals (Tanaka et al., 2005), whereas relaxin-3 projections into the septohippocampal system/pathway modulates memory (Ma et al., 2009). Stimulating the relaxin-3 signaling system is beneficial for reducing anxiety-like behavior (Zhang et al., 2015a). RXFP3 is expressed in neurons in the paraventricular region of the hypothalamus (Liu et al., 2003; Ma et al., 2007; Smith et al., 2010), and stimulation of these neurons with relaxin-3 agonists or antagonists regulates appetite in rodent models. Satiated rats treated with relaxin-3 increase their food intake, an effect that is reduced by relaxin-3 antagonist pre-treatment (Kuei et al., 2007). In mouse models where the feeding drive is high, including mild food deprivation and palatable food paradigms, treatment with relaxin-3 antagonists reduce food intake (Smith et al., 2014). Furthermore, studies have shown an antagonist is able to block stress-induced binge-eating in female rats (Calvez et al., 2016b) and revealed sex-specific effects of relaxin-3 in control of feeding (Calvez et al., 2016a). Antagonizing the relaxin-3/RXFP3 system is also beneficial for reducing alcohol seeking in rat addiction models (Ryan et al., 2013). Thus, there is strong pharmaceutical potential for relaxin-3 analogs for treating a wide range of neurological disorders (Kumar et al., 2017).

**Figure 1:**
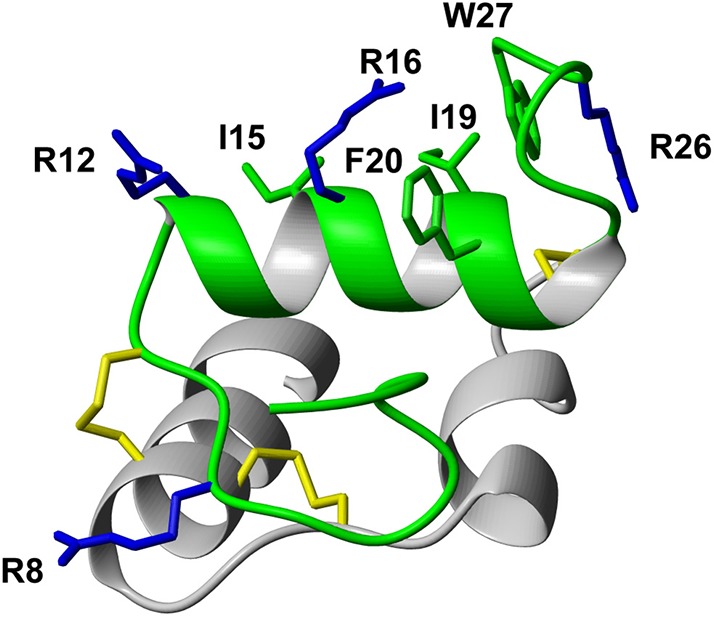
Relaxin-3 solution NMR structure. The A-chain (gray) and B-chain (green) are cross-braced by one intramolecular disulfide bond in the A-chain and two intermolecular disulfide bonds (yellow). Important relaxin-3 B-chain residues involved in binding and activation of RXFP3 are shown in sticks and colored blue and green for basic and hydrophobic residues, respectively.

Following the determination of the solution NMR structure of the two-chain native relaxin-3 peptide (Figure 1) (Rosengren et al., 2006), detailed structure-activity relationship studies have been undertaken (Patil et al., 2017). Studies on chimeric variants using combinations of A- and B-chains from different members of the insulin/relaxin family identified the relaxin-3 B-chain as the key region for interacting with RXFP3 (Liu et al., 2005). The first nine residues in the α-helix in the A-chain can be truncated (Hossain et al., 2008) and the A-chain disulfide bond removed (Shabanpoor et al., 2012) without severely affecting binding and potency at RXFP3. However, the overall structure of such analogs is compromised, hence the function of the A-chain is likely to provide a structural support to the B-chain (Hossain et al., 2008). Point mutations throughout the relaxin-3 B-chain have identified residues that contribute to binding and activation of RXFP3 (Kuei et al., 2007). ArgB8, ArgB12, IleB15, ArgB16, IleB19, and PheB20, which largely form a continuous binding surface on the B-chain helix, are important for binding. In addition, the two C-terminal residues (ArgB26 and TrpB27) are crucial for the activation of RXFP3 and their removal results in the creation of an antagonist (Kuei et al., 2007) (Figure 1).

**Figure 2:**
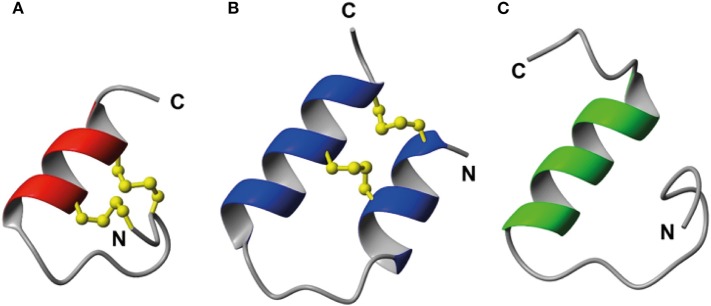
Structural comparison of **(A)** apamin (red) and **(B)** VhTI (blue) with **(C)** the relaxin-3 B-chain (green). The apamin and VhTI scaffolds are stabilized by two disulfide bonds and include α-helices between residues 9–18 and 3–25, respectively.

As the key residues are located in the B-chain the B-chain alone can act as an agonist, albeit with significant lower affinity (Liu et al., 2005). A high affinity single chain antagonist based on a truncated B-chain in which the five C-terminal residues are exchanged for an Arg (R3 B1-22R) has been developed (Haugaard-Kedström et al., 2011). In this analog the non-native Arg23 forms significant interactions with RXFP3, compensating for the loss of affinity due to the compromised structure (Haugaard-Kedström et al., 2018; Wong et al., 2018). For single chain agonists the affinity can instead be improved by reintroducing helical structure in the absence of the A-chain through “helical stapling” using dicarba bonds (Hojo et al., 2016; Jayakody et al., 2016). Linear peptides such as the R3 B1-22R antagonist and the relaxin-3 B-chain generally are readily broken down in serum and require redesign to be feasible drug leads. One such redesign concept is “molecular grafting” where essential residues for bioactivity are introduced at topologically equivalent positions of a stable scaffold with appropriate structure, as a way to reduce enzymatic susceptibility of the binding motif (Wang and Craik, 2018). The key binding features of relaxin-3 are centered around the B-chain helix, thus an appropriate grafting scaffold for targeting RXFP3 would have to include a helix of similar length to native relaxin-3. Several α-helical scaffolds have been evaluated for peptide based drug development, including apamin (Li et al., 2009), the zinc finger Zif268 (Mccoll et al., 1999) and MCoTI-I (Ji et al., 2013). Apamin is an 18 amino acid long natural product derived from bee venom and consists of an α-helical region, which is stabilized by two disulfide bonds between the helix and the N-terminal loop region (Figure 2). Apamin has previously been used as a grafting scaffold for the development of p53 inhibitors, and ligands for the both the estrogen receptor and p32 (Li et al., 2009; Phan et al., 2010; Zhang et al., 2015b). Another scaffold identified here as interesting for relaxin-3 grafting is a 34 amino acid residue trypsin inhibitor from the seeds of *Veronica hederifolia* (VhTI), which has a helix-loop-helix fold (Figure 2). The structure is stabilized by two disulfide bonds cross-linking the helices at adjacent turns (Conners et al., 2007).

In this study we designed and synthesized seven grafted relaxin-3 agonists and antagonists by exploiting the two disulfide-stabilized α-helical peptide scaffolds, apamin and VhTI (Figure 2). The analogs were studied by solution NMR spectroscopy, and their affinity and potency at RXFP3 determined. The grafted peptides were able to adopt the native helical structure, and selected peptides retained RXFP3 affinity and activity. Furthermore, they had significantly increased serum stability, thus are promising ligands for further development of RXFP3 selective agonists and antagonists.

## Experimental Section

All amino acids were purchased from GL Biochem (Shanghai, China). Fmoc-Trp(Boc) Tentagel S-PHB resin (0.23 mmol/g) and PAL-PEG-PS resins (0.20 mmol/g) were purchased from Rapp Polymere (Tuebingen, Germany) and Applied Biosystems (Victoria, Australia), respectively. All solvents and chemicals were purchased from Merck (Victoria, Australia) and were of peptide synthesis grade.

## Peptide Synthesis

Linear peptides were assembled using a CS 336X (CSBio) or an Alstra microwave peptide synthesizer (Biotage). Using Fmoc-based solid phase peptide methodology, agonists were synthesized on resins preloaded with the C-terminal Trp residue. Apa+R3B, Apa+R3B[V18Aib,T21Aib], VhTI+R3B, and VhTI+R3B[G11,R12] were assembled on Fmoc-Trp(Boc)-Peg-PS resin with 4 eq. Fmoc-protected amino acids, 4 eq. HBTU and 4 eq. diisopropylethylamine (DIPEA). VhTI+R3B[R12] on the other hand was assembled on Fmoc-Trp(Boc)-Tentagel S PHB resin with 5 eq. Fmoc-protected amino acids. Apa+R3 B1-22R and VhTI+R3 B1-22R were assembled on Rink Amide and Pal-Peg-PS resins, respectively. Val, Ile, Thr and Arg residues were routinely double coupled during chain assembly. Fmoc deprotection was carried out using 20% piperidine in DMF. The linear peptides were cleaved off the resin using TFA:TIPS:DODT:H_2_O (92.5: 2.5: 2.5: 2.5) for 2 h, followed by filtration. The TFA was evaporated under vacuum and the peptides were precipitated using ice-cold diethyl ether. Precipitated peptides were redissolved in 50/50 buffer A (0.05% TFA in H_2_O) and buffer B (90% acetonitrile and 0.045% TFA in H_2_O), before lyophilisation. The linear peptides were purified using C18 reversed phase columns on a Prominence HPLC system (Shimadzu) with a gradient of buffer A and buffer B. Characterization of all analogs were conducted using electro-spray ionization mass spectrometry on an API2000 (AB Sciex). Analogs were analyzed for purity using analytical RP-HPLC at 1% gradient and confirmed as >95% pure.

## Oxidation of Apamin Grafted Peptides

The apamin grafted peptides were oxidized using random oxidation. The linear peptides were dissolved in 20 mM Tris HCl, pH 8 at 0.25 mg/ml and stirred for 72 h at room temperature, according to previous reported conditions (Volkman and Wemmer, 1997).

## Oxidation of VhTI Grafted Peptides

The linear VhTI grafted peptides were either oxidized using a random oxidation procedure or by regioselective disulfide bond formation. For random oxidation, 0.1 mg/ml linear peptide was dissolved in 50 mM Tris HCl, pH 8.6 and stirred at room temperature overnight. For regioselective disulfide bond formation, acid stable Acm orthogonal protecting groups were used for one cysteine pair. The first disulfide bond was formed by dissolving the Acm-protected linear peptide in 50/50 acetonitrile/H_2_O at a concentration of 0.33 mg/ml followed by addition of 0.1 ml/mg 2-DPDS dissolved in methanol. The reaction was carried out over night at room temperature before purification by RP-HPLC. In order to form the second disulfide bond, the peptide was dissolved (0.5 mg/ml) in 50% acetic acid in H_2_O and degassed with nitrogen for 5 min. 0.1 M HCl (0.1 ml/mg) was added to the peptide, followed by I_2_ dissolved in 50% acetic acid until the solution turned light brown. The peptide solution was degassed briefly and left under stirring at room temperature for 2 h in the dark, before the reaction was stopped using 1 M ascorbic acid, with sufficient amount added until the solution changed to colorless. Buffer A was added to dilute the peptide solution before loading onto the RP-HPLC column for purification. The final peptides were characterized using analytical RP-HPLC, LC-MS, and NMR spectroscopy.

## Solution NMR Spectroscopy

0.5–1 mg of grafted peptides were dissolved in 90% H_2_O and 10% D_2_O and subjected to solution NMR spectroscopy studies. Two dimensional (2D) ^1^H homonuclear total correlation spectroscopy (TOCSY) with a mixing time of 80 ms, double-quantum filtered correlation spectroscopy (DQF-COSY) and nuclear Overhauser effect spectroscopy (NOESY) with a mixing time of 200 ms data were recorded at 298K and 600 MHz using an Advance spectrometer equipped with a cryoprobe (Bruker). For the apamin variants, additional ^13^C HSQC data were recorded at natural abundance, allowing determination of ^13^C chemical shifts. Amide proton temperature coefficients were determined from TOCSY data sets recorded at temperatures 288–308 K. All data were processed using Topspin and referenced to the solvent signal.

## Structure Determination

All spectra were analyzed using CARA (Keller, 2004). Resonance assignments were achieved using homonuclear sequential assignment strategies (Wüthrich, 1986) and peaks in the NOESY spectra integrated and automatically assigned and translated into distance constraints using the software package CYANA (Guntert, 2004). Backbone dihedral angels (φ and ψ) where derived from chemical shifts using TALOS-N (Shen and Bax, 2013). Hydrogen bonds were identified from temperature coefficients and included as constraints for amides with a temperature dependence Δδ_HN_/ΔT > −4.6 ppb/K if a suitable hydrogen bond acceptor could be identified in preliminary structures (Cierpicki and Otlewski, 2001). The structure calculations were performed using torsion angle dynamics within CYANA (Guntert, 2004). The 20 lowest target function models from a total of 50 structures calculated were chosen to represent the solution structure and analyzed using MOLPROBITY (Chen et al., 2010).

## Cell Based RXFP3 Binding and Activity Assays

The affinity and activity of the grafted peptides were determined as previously described (Haugaard-Kedström et al., 2015). Briefly, for competition binding studies CHO-K1 cells stably expressing RXFP3 were incubated with 5 nM Eu-DTPA-R3 B1-22R and increasing concentrations of peptide analogs. Unlabeled R3 B1-22R was used as reference compound. The europium was detected by the addition of enhancement solution and fluorescence recorded using an excitation of 340 nm and emission reading at 614 nm. For agonists the ability of analogs to inhibit cAMP accumulation were tested in CHO-K1 cells stably expressing RXFP3 transfected with a pCRE-β-galactosidase reporter plasmid. Cells were stimulated with 5 μM forskolin and incubated with increasing concentrations of relaxin-3 analogs as previously described (Shabanpoor et al., 2012). Each concentration point was conducted in triplicates and whole experiment was repeated three times independently. Data are expressed as mean ± SEM and analyzed using Prism 8 (GraphPad).

## *In vitro* Serum Stability

The *in vitro* serum stability was determined by peptide spiking, as previously described (Hossain et al., 2015). Briefly, 570 μl human male pooled serum was spiked with 30 μl peptide stock solution (0.05 μg/μl in H_2_O). 100 μl samples were taken out at different time points and quenched with 900 μl ammonium acetate (0.1 M, pH 3). Samples were left on ice for 30 min before being extracted using solid phase extraction cartridges (Oasis HLB 3cc, Waters). Eluted samples were then lyophilized and reconstituted in 1% formic acid. The amount of intact peptide is reported relative to the amount of peptide available at timepoint 0 hr. Quantification was carried out using multiple reaction monitoring on a triple-quad LC-MS (API2000, AB Sciex) and the data analyzed using one phase decay in Prism 7.

## Results and Discussion

### Peptide Design and Synthesis

The native relaxin-3 B-chain is a weak agonist (Liu et al., 2005), but using helical staples significantly improves activity (Hojo et al., 2016; Jayakody et al., 2016). Stapling has also been shown to facilitate peptide delivery to the brain via the intranasal route (Marwari et al., 2019). Given the success with employing molecular grafting as a drug design approach (Li et al., 2009; Phan et al., 2010; Zhang et al., 2015b; Wang and Craik, 2018), we envisioned that single chain RXFP3 agonists and antagonists with improved stability, and possibly also affinity and efficacy, could alternatively be developed by transferring key residues from the two-chain relaxin-3 onto a stable single chain scaffold with appropriate structure. The RXFP3 binding motif (ArgB8, ArgB12, IleB15, ArgB16, IleB19, and PheB20) is predominantly situated on the solvent exposed side of the α-helix located in the native relaxin-3 B-chain (Rosengren et al., 2006). The C-terminal ArgB26 and TrpB27 are located in the highly flexible C-terminal part of the B-chain, which extend from the helical segment and interact with the central pore of the transmembrane receptor helix bundle (Bathgate et al., 2013; Hu et al., 2016; Wong et al., 2018). These residues could thus potentially be grafted onto an α-helical scaffold and retain the native bioactive secondary structure. Table 1 outlines seven novel analogs that were designed based on the apamin and VhTI scaffolds and compares them to reference compounds. Analogs **1** (native relaxin-3), **2** (relaxin-3 B-chain) and **8** (single chain relaxin-3 antagonist) are included as reference compounds for comparison with the novel grafted analogues' affinity and potency at RXFP3. Analog **3** was designed using the apamin scaffold and the relaxin-3 B-chain binding and activating motifs. In order to further support the helical propensity of analog **3**, analog **4** also includes aminoisobutyric acid (Aib) amino acids, known to induce helicity due to the stereochemical constraint of an additional methyl group at the Cα carbon (Mahalakshmi and Balaram, 2006). Val18 and Thr21 of relaxin-3 B-chain were substituted with Aib in analog **4**, as those residues are not important for activity of relaxin-3 (Kuei et al., 2007), and a recent study has shown that Aib can be incorporated at these positions without adversely affecting binding of R3 B1-22R to RXFP3 (Haugaard-Kedström et al., 2018). Analog **5** is based on the VhTI scaffold and incorporates residues equivalent to ArgB8, IleB15, ArgB16, PheB20, and the C-terminal tail. The equivalent position of ArgB12 is a proline in VhTI, and may be structurally important, thus was retained in this variant. In analog **6**, the Pro was replaced to include ArgB12 and the conservative change of Leu to Ile was also included to match IleB19 in relaxin-3. In analog **7**, the entire binding cassette was shifted one residue closer to the C-terminus to change the positioning of the motif on the helical surface. Finally, we have previously shown that by truncating the last five native C-terminal residues in the B-chain and replacing them with an arginine, a single chain RXFP3 antagonist can be generated (Haugaard-Kedström et al., 2011). This formed the basis for constructing two new antagonist variants **9** and **10** that were grafted onto apamin and VhTI, respectively. All peptides were synthesized using solid phase peptide synthesis and purified by RP-HPLC to obtain peptides with high degree of purity (>95%). HPLC traces and MS data of oxidized analogs are included in Figures S1, S2.

**Table 1:** Amino acid sequences of grafted RXFP3 agonists (3–7) and antagonists (9–10).

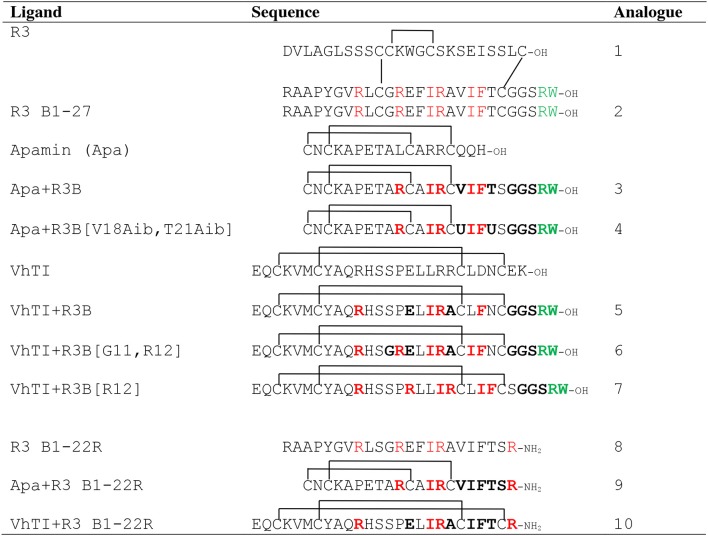

*U, aminoisobutyric acid. Bold letters indicate corresponding relaxin-3 B-chain peptide grafted onto the scaffold. Red letters; residues important for RXFP3 binding. Green letters; residues important for RXFP3 activation*.

### NMR Structural Studies of Peptide Analogs

**Figure 3:**
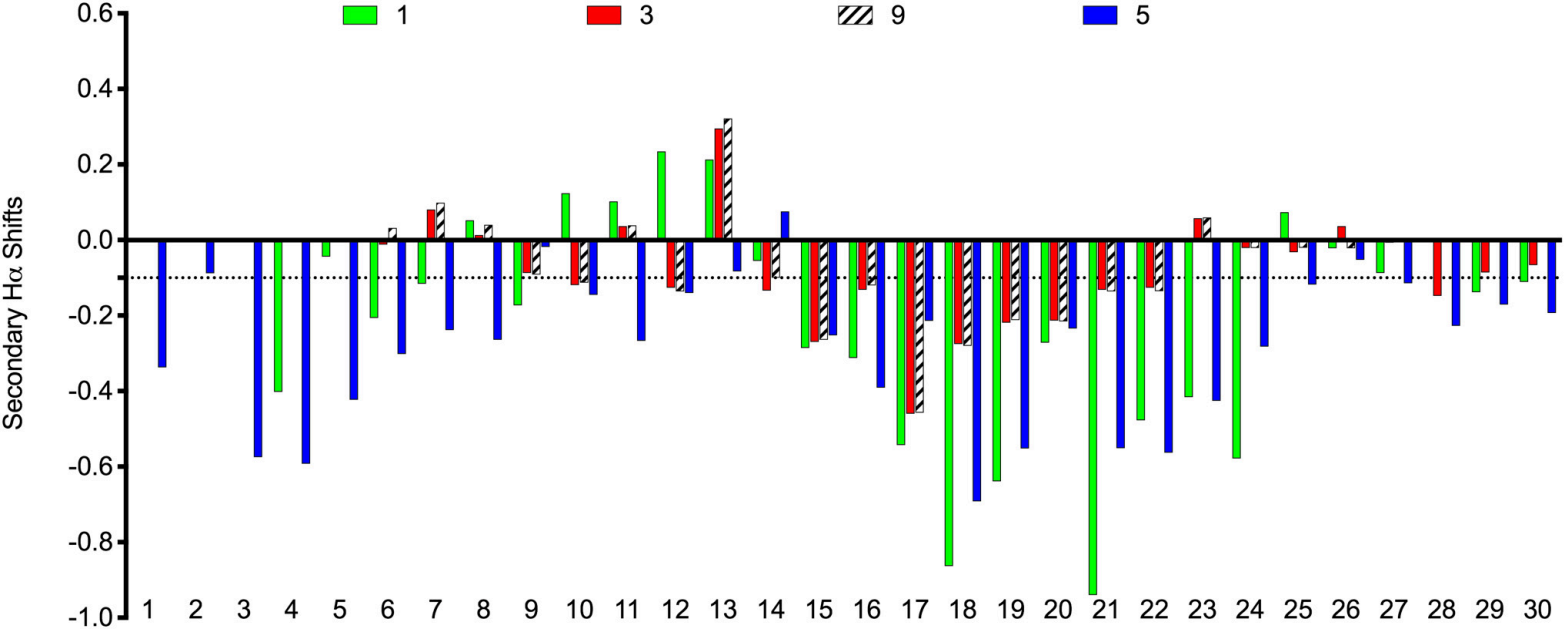
Comparison of secondary Hα chemical shifts for apamin and VhTI based relaxin-3 analogs and native relaxin-3. The stretch of negative values from 15 to 24 correspond to the relaxin-3 B-chain helix. Residue numbers on the X-axis relate to the longest peptide sequence, analog 5, while the sequences are aligned so that the key grafted residues are at the same position in each analog.

The scaffolds were chosen to mimic the desired structure of relaxin-3. Whether, the peptide analogs had in fact been able to fold into their expected helical conformation was assessed by solution NMR spectroscopy. The NMR data for analogs **3, 4, 5, 9**, and **10** were of good quality with sharp lines and excellent signal dispersion, consistent with a well-ordered structure. In contrast, the NMR data for the VhTI grafted analogs, **6** and **7**, were of poor quality with limited dispersion, and in the case of analog **7** broad lines, consistent with unstructured or misfolded peptides. NOESY data for all analogs are included as supplementary information, highlighting the difference in spectral quality (Figures S3–S9). Resonance assignment of the NMR data were completed for **3**, **4**, **5**, and **9** using homonuclear sequential assignments strategies. These peptides were all synthesized using random oxidation, but their correct disulfide arrangement could be confirmed by the NMR analysis. Similar oxidation protocols have been used in previous studies and resulted in the native disulfide conformation for apamin (Le-Nguyen et al., 2007; Li et al., 2009). Poor quality of the NMR data of analogs **6** and **7** were observed even though these peptides were synthesized using regioselective disulfide bond formation by first forming the Cys7-Cys21 disulfide bond followed by the Cys3-Cys25 disulfide for analog **6** and vice versa for analog **7**. This approach ensured the fold was not compromised because of the formation of an incorrect disulfide isomer. Given analogs **6** and **7** are unable to adopt a native fold, the VhTI scaffold is clearly more sensitive to amino acid changes than the apamin structure. Secondary Hα chemical shifts are excellent indicators of secondary structure, with stretches of negative values being indicative of helical structure while stretches of positive values being consistent with β-sheet (Wishart et al., 1995). A comparison of the secondary Hα shifts observed for the B-chain in native relaxin-3 (**1**) and analogs **3**, **5**, and **9**, highlight that analog **5** most closely mimic the negative values of relaxin-3 throughout the entire helical region (Figure 3). The apamin based analogs also adopt a helical conformation as evident from negative secondary shifts, but the C-terminal part of the helix appears to be less ordered that in relaxin-3. For the linear single chain antagonist peptide R3 B1-22R (Haugaard-Kedström et al., 2011), the secondary shifts are close to zero, thus, grafting of important relaxin-3 residues onto apamin or VhTI is a clearly an effective strategy for restoring helical secondary structure of all these analogs.

**Figure 4:**
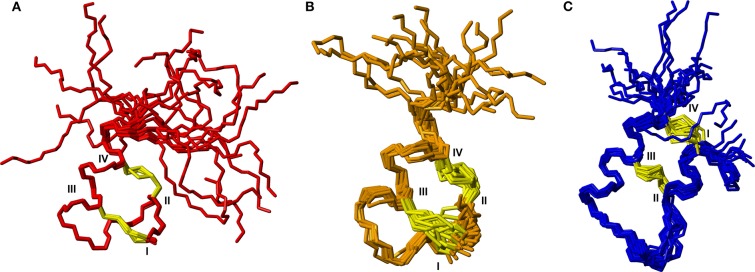
Backbone superposition of the 20 best structures calculated based on NMR spectroscopy data for **(A)** analog **3**, **(B)** analog **9**, and **(C)** analog **5**. Cystine side chains are shown in yellow. Cystine positions in the sequences are indicated by roman numerals.

To further characterize the structures of the grafted analogs, the full 3D structures were calculated based on NMR data for analogs **3**, **5**, and **9**. Interproton distance restraints were derived from cross-peak intensities in the NOESY data, hydrogen bond donors were identified from temperature coefficients and for the apamin variants where ^13^C chemical shifts could be determined from natural abundance HSQC data, TALOS-N was used to derive backbone dihedral angle restraints. These data were used as input for torsion angle dynamics calculations using CYANA. From a total of 50 structures generated, 20 structures with good CYANA target functions and stereochemistry based on MOLPROBITY (Chen et al., 2010) analyses were chosen to represent the solution structures of the variants. Structural superpositions of these are shown in Figure 4. From here it is clear that the disulfide bonded cores are well defined, while the C-terminal tail is disordered, consistent with the secondary shifts. The helical regions observed in the analogs match the helices seen in the native structures of the apamin and VhTI scaffolds. The structural statistics are given in Table S1.

### Assessment of Affinity and Activity of Peptide Analogs at RXFP3

**Figure 5:**
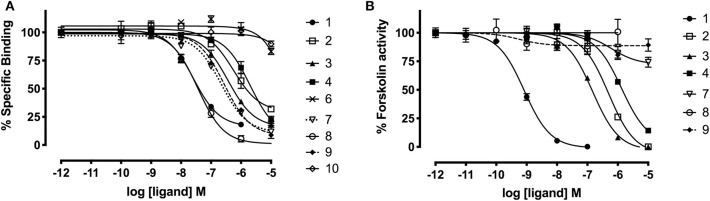
Affinity and activity of relaxin-3 grafted agonists and antagonist at RXFP3. **(A)** Competitive binding curves for grafted peptides using CHO cells stably expressing RXFP3 and the fluorescent tracer Eu^3+^-R3B1-22R. **(B)** Activity of grafted agonists using a reporter assay in CHO cells stably expressing RXFP3. Activation is measured as a reduction in forskolin induced cAMP activity. Data are shown as mean ± SEM of triplicate determinations from a minimum of three independent experiments.

Since grafting of the relaxin-3 epitope onto apamin and VhTI showed an improvement in the overall structure, we investigated whether this change translated into improvements in binding and activation of RXFP3. The binding affinity of the grafted peptides were determined using CHO cells stably expressing RXFP3 by measuring increasing concentrations of the grafted peptides' ability to compete for the binding site with europium labeled R3 B1-22R (Haugaard-Kedström et al., 2015). Activation was measured by assessing the ability of the analogs to inhibit cAMP accumulation induced by forskolin in a reporter assay, as RXFP3 couples to an inhibitory G-protein. The binding and activation data are presented in Figure 5 and summarized in Table 2.

**Table 2:** Binding affinities and cAMP activity of grafted analogs.

**Agonists**	**Analog**	**Binding affinity** **pK_**i**_ ± SEM [logM]^**a**^**	**cAMP activity** ** pEC_**50**_ ± SEM [logM]**
R3 acid	**1**	7.69 ± 0.12 (3)	9.08 ± 0.07 (5)
R3 B1-27	**2**	5.91 ± 0.21 (3)^b^	5.93 ± 0.02 (3) ^b^, ^i^
Apa+R3B	**3**	6.65 ± 0.18 (3)^b^, ^g^	6.83 ± 0.07 (3) ^b^, ^e^
Apa+R3B [V18Aib,T21Aib]	**4**	5.70 ± 0.10 (3)^b^	5.88 ± 0.06 (3) ^b^
VhTI+R3B	**5**	<5	ND
VhTI+R3B[G11, R12]	**6**	<5	ND
VhTI+R3B[R12]	**7**	6.91 ± 0.01 (2)^d^, ^f^	<5
**Antagonists**			
R3 B1-22R	**8**	7.69 ± 0.18 (4)^e^	No activity
Apa+R3 B1-22R	**9**	6.76 ± 0.03 (3)^c^, ^f^, ^h^	No activity
VhTI+R3 B1-22R	**10**	<5	ND

a *pK_i_-values are calculated based on the K_d_ 27.9 ± 9.4 nM for Eu-R3 B1-22R. Numbers in parentheses indicate the times each experiment has been repeated as independent triplicates*.

b *p < 0.001*,

c p < 0.01, and

d *p < 0.05 compared to R3 acid*.

e *p < 0.001*,

f p < 0.01 and

g *p < 0.05 compared to R3 B1-27*.

h *p < 0.05 compared to R3 B1-22R*.

i *, from ref (Hojo et al., 2016). ND, not determined*.

The apamin grafted agonist analog **3**, which included ArgB12, IleB15, ArgB16, IleB19, and PheB20, showed both increased potency (pEC_50_ = 6.83) and affinity (pK_i_ = 6.65) compared to the relaxin-3 B-chain (analog **2**) (pEC_50_ = 5.93 and pK_i_ = 5.91), confirming the importance of a helical secondary structure in the relaxin-3 B-chain. This is consistent with previous findings where inducing helicity of the single-chain agonist using dicarba stapling efficiently improved binding affinity and activation of RXFP3, compared to the unstructured relaxin-3 B-chain (Hojo et al., 2016; Jayakody et al., 2016). The NMR analysis showed that in analog **3** the helix is shorter than in native relaxin-3, suggesting that additional modifications further supporting the C-terminal part of the helix may be beneficial. Intriguingly, analog **4** which was designed with this in mind included Aib residues at positions ValB18 and ThrB21, but rather than improving activity this decreased both the affinity (pK_i_ = 5.70) and activation (pEC_50_ = 5.88) of RXFP3 to comparable levels of the native relaxin-3 B-chain (analog **2)**. The drop in binding affinity was surprising, given the secondary Hα shifts confirmed the addition of Aib indeed increased the degree of helicity of analog **4** compared to analog **3** (Figure S10). This suggest that either a degree of flexibility is favorable to allow adaptation of the binding conformation, or that the Val and Thr side chains do contribute to binding in the context of this analog.

**Figure 6:**
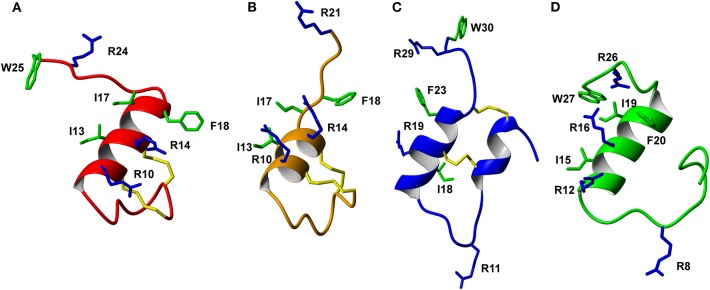
Comparison of the solution NMR structures of **(A)** analog **3**, **(B)** analog **9**, **(C)** analog **5**, and **(D)** analog **1** (B-chain only). The grafted apamin analogs **(A,B)** and grafted VhTI **(C)** analog show overall secondary structure similar to the native H3 relaxin B-chain **(D)**. The grafted RXFP3 binding and activating residues are shown, highlighting differences in the positioning of the receptor binding motif in the various analogs.

The trypsin inhibitor scaffold VhTI was also investigated by grafting the corresponding relaxin-3 residues ArgB8, IleB15, ArgB16, PheB20, and the flexible C-terminal residues GlyB23, GlyB24, SerB25, ArgB26, and TrpB27. In analog **5** ArgB12 was omitted, as the corresponding residue in VhTI was a Pro, and based on the structure, could potentially be important for the fold. In addition, ArgB20 of VhTI was mutated to an Ala to prevent charge and/or steric clashes. Analog **5** was well-structured, closely replicating the secondary shifts of native relaxin-3, however the affinity of **5** for RXFP3 was poor (pK_i_ < 5). To address the absence of ArgB12 a second version was designed to include both GlyB11 and ArgB12 from native relaxin-3 (analog **6**). The affinity of **6** was however not improved compared to **5**, and the NMR structural analysis showed that this version was not able to adopt the correct fold. We conclude that indeed the Pro is likely a prerequisite for correct folding of the helix-turn-helix motif in VhTI. Comparing the NMR structures of our grafted analogs with the relaxin-3 B-chain we noted that in both apamin based variants the binding motif was positioned on top of the helical structure, extending away from the stabilizing core, in a very similar fashion to the arrangement in relaxin-3. In contrast, in the folded VhTI variant (analog **5**) the motif is rotated slightly around, which may prevent full access of key grafted residues, including IleB15 (Figure 6). Thus, based on the determined structure of analog **5**, a third VhTI grafted agonist variant, analog **7**, was designed. Here the positions of ArgB12, IleB15, IleB19, and PheB20 were moved one residue toward the C-terminus to fit the positioning of the native relaxin-3 relative to the helical surface, which also allowed the structural Pro in native VhTI to be retained. This modification did improve affinity (pK_i_ = 6.91) but the peptide was not able to activate RXFP3 (pEC_50_ = <5). Furthermore, structural analysis using NMR showed the peptide was largely unstructured rather than being able to retain the VhTI fold, which would explain the lack of potency. We have previously shown that the single chain antagonist R3 B1-22R is able to bind efficiently to RXFP3 without being structured in solution (Haugaard-Kedström et al., 2011), and in fact the flexibility is favored for being able to adapt to bind efficiently to RXFP3 in a slightly different conformation relative to native relaxin-3 (Haugaard-Kedström et al., 2018; Wong et al., 2018).

In addition to the agonist grafted peptides, we also designed and synthesized two molecular grafted antagonists based on apamin and VhTI (**9** and **10**). The apamin grafted antagonist (**9**) retained binding to RXFP3, but the pK_i_ was 6.76, which is about ~10-fold weaker compared to the linear antagonist R3 B1-22R. The NMR structural analysis confirmed that the apamin scaffold does confer helical structure, but that the C-terminal part of the peptide after the last cysteine, which includes the key antagonist binding residue ArgB23, remains highly flexible. Again this is consistent with detailed structure activity analysis of R3 B1-22R, which suggest a degree of flexibility is a prerequisite for optimal binding (Haugaard-Kedström et al., 2018; Wong et al., 2018). The VhTI grafted antagonist was based on the first version of the VhTI agonist and retained the native Pro residue from VhTI instead of ArgB12. The relative importance of ArgB12 for binding to RXFP3 in antagonists is significantly less than in native relaxin-3 (Haugaard-Kedström et al., 2018; Wong et al., 2018), thus we did not anticipate this being detrimental to affinity. However, analog **10** showed very poor binding to RXFP3 (Table 2, Figure 6), which again is likely related to the positioning of the binding residues on the helical surface. Given the inability of our next generation VhTI agonist variants (analogs **6** and **7**) to fold correctly we did not pursue this scaffold further in terms of redesign of additional antagonists.

Taken together for the design of both agonists and antagonists, the apamin framework appears more adaptable and able to accommodate the non-native amino acids while remaining structurally stable. The VhTI scaffold is more sensitive to sequence changes and it may also be too large and hence interfere with the interaction between the key relaxin-3 residues and the RXFP3 binding site. It is clear that grafting onto different disulfide scaffolds can rescue/restore the secondary structure of relaxin-3 based peptides, but it does not always correlate with an improvement in activity. Similar findings have been reported for synthetic GLP-1R agonists, where peptides stabilized by a disulfide linked conotoxin scaffold showed improved helicity, but the potency did not reflect the gain in helicity (Swedberg et al., 2016).

### Stability of Grafted Peptide Analogs in Serum

**Figure 7:**
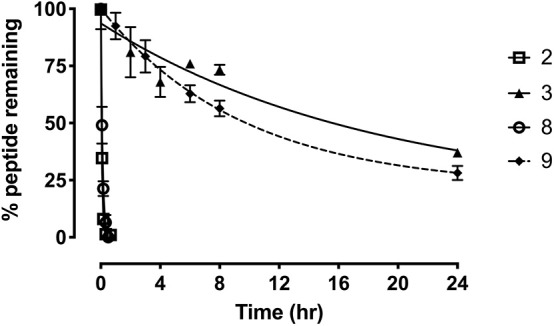
Serum stability of the single-chain R3 B1-27 (**2**), Apa+R3B (**3**), R3 B1-22R (**8**), and Apa + R3 B1-22R (**9**) as measured in % peptide remaining in serum at various time points. Data presented as mean ± SEM from at least three replicates.

To further evaluate the potential of the most promising grafted peptides, the half-life in serum was determined for the apamin based analogs **3** and **9** and compared to their linear counterparts (Figure 7). The serum stability of **3** showed a remarkable increase (T_1/2_ = 12.8 h) compared to the linear R3 B1-27 (T_1/2_ = 3.1 min). Similarly, stability of the grafted antagonist **9** (T_1/2_ = 6.6 h) was significantly improved compared to the linear antagonist R3 B1-22R (T_1/2_ = 4 min). This equates to a 250-fold and 75-fold improvement in serum stability for the agonist and antagonist, respectively. The change in peptide stability is most likely due to increasing structural constraints, as unstructured peptides are generally less stable compared to their grafted analogs (Wang et al., 2014). Apamin itself is known to be a highly stable peptide (Oller-Salvia et al., 2013) and improving peptide secondary structure has previously been shown to improve stability using both small (Shepherd et al., 2005) and large scaffolds (Ji et al., 2013; Fujiwara et al., 2016), leading to better functional outcomes (Weston et al., 2004).

## Conclusions

In conclusion, relaxin-3 residues involved in binding and activity at RXFP3 were successfully grafted onto two different scaffolds, apamin and VhTI, to form potential agonist and antagonist analogs of relaxin-3. Native relaxin-3 is a two-chain peptide with two intermolecular and one intramolecular disulfide bond, which makes its synthesis complex and costly, thus a single chain peptide that is able to adopt the correct structure and activity is highly desirable. The relaxin-3 agonist based on the apamin scaffold showed improved structure, binding affinity and potency for RXFP3 compared to the linear relaxin-3 B-chain. Although one variant of the VhTI scaffold was also able to restore the α-helix structure of the native B-chain of relaxin-3, none had the desired pharmacology. Similarly, for the antagonist design, the use of the apamin scaffold improved helical structure, but relative to the single chain antagonist R3 B1-22R, a reduction in binding was observed. Nonetheless, both the apamin based variants showed remarkable improvement in serum stability, which is critical for *in vivo* studies. Using apamin as a scaffold is particularly promising given this peptide is known to be able to pass the blood brain barrier and has been used as a shuttle to deliver therapeutic agent into the brain (Wu et al., 2014). Given RXFP3 is a neuropeptide receptor predominantly expressed in the brain apamin based analogs may, for the first time, be able to allow efficient systemic delivery of RXFP3 modulators for studying the roles of this signaling system.

## Data Availability Statement

The raw data supporting the conclusions of this article will be made available by the authors, without undue reservation, to any qualified researcher.

## Author Contributions

LH-K and KR design of project. HL, MP, RC, and LH-K peptide synthesis and chemical analysis. MP and KR NMR spectroscopy data acquisition and analysis. MP, HL, and KR 3D structure determination and analysis. RB cell based RXFP3 assays. HL and RB statistical analysis. AS, HL, and LH-K serum stability assays. HL, LH-K, and KR manuscript drafting. HL, AS, RC, RB, LH-K, and KR manuscript editing and finalization. KR project administration and supervision. KR and RB funding.

### Conflict of Interest

RB, LH-K, and KR are inventors on Australian Patent 2010904046 and United States patent application 13/821726, Modified Relaxin B Chain Peptides. The remaining authors declare that the research was conducted in the absence of any commercial or financial relationships that could be construed as a potential conflict of interest. The reviewer JM declared a past co-authorship with one of the authors RB to the handling editor.

## References

[B1] Bathgate R. A. Oh M. H. Ling W. J. Kaas Q. Hossain M. A. Gooley P. R.. (2013). Elucidation of relaxin-3 binding interactions in the extracellular loops of RXFP3. Front. Endocrinol. 4:13. 10.3389/fendo.2013.00013 23440673 PMC3579193

[B2] Bathgate R. A. Samuel C. S. Burazin T. C. Layfield S. Claasz A. A. Reytomas I. G.. (2002). Human relaxin gene 3 (H3) and the equivalent mouse relaxin (M3) gene. Novel members of the relaxin peptide family. J. Biol. Chem. 277, 1148–1157. 10.1074/jbc.M107882200 11689565

[B3] Calvez J. De Avila C. Guevremont G. Timofeeva E. (2016a). Sex-specific effects of chronic administration of relaxin-3 on food intake, body weight and the hypothalamic-pituitary-gonadal axis in rats. J. Neuroendocrinol. 28. 10.1111/jne.12439 27791297

[B4] Calvez J. De Avila C. Matte L. O. Guevremont G. Gundlach A. L. Timofeeva E. (2016b). Role of relaxin-3/RXFP3 system in stress-induced binge-like eating in female rats. Neuropharmacology 102, 207–215. 10.1016/j.neuropharm.2015.11.014 26607097

[B5] Chen V. B. Arendall W. B. III. Headd J. J. Keedy D. A. Immormino R. M. Kapral G. J.. (2010). MolProbity: all-atom structure validation for macromolecular crystallography. Acta Crystallogr. D Biol. Crystallogr. 66, 12–21. 10.1107/S0907444909042073 20057044 PMC2803126

[B6] Cierpicki T. Otlewski J. (2001). Amide proton temperature coefficients as hydrogen bond indicators in proteins. J. Biomol. NMR 21, 249–261. 10.1023/A:1012911329730 11775741

[B7] Conners R. Konarev A. V. Forsyth J. Lovegrove A. Marsh J. Joseph-Horne T.. (2007). An unusual helix-turn-helix protease inhibitory motif in a novel trypsin inhibitor from seeds of Veronica (*Veronica hederifolia* L.). J. Biol. Chem. 282, 27760–27768. 10.1074/jbc.M703871200 17640870

[B8] Fujiwara D. Kitada H. Oguri M. Nishihara T. Michigami M. Shiraishi K.. (2016). A cyclized helix-loop-helix peptide as a molecular scaffold for the design of inhibitors of intracellular protein-protein interactions by epitope and arginine grafting. Angew. Chem. Int. Ed. Engl. 55, 10612–10615. 10.1002/anie.201603230 27467415

[B9] Guntert P. (2004). Automated NMR structure calculation with CYANA. Methods Mol. Biol. 278, 353–378. 10.1385/1-59259-809-9:353 15318003

[B10] Haugaard-Kedström L. M. Lee H. S. Jones M. V. Song A. Rathod V. Hossain M. A.. (2018). Binding conformation and determinants of a single-chain peptide antagonist at the relaxin-3 receptor RXFP3. J. Biol. Chem. 293, 15765–15776. 10.1074/jbc.RA118.002611 30131342 PMC6187623

[B11] Haugaard-Kedström L. M. Shabanpoor F. Hossain M. A. Clark R. J. Ryan P. J. Craik D. J.. (2011). Design, synthesis, and characterization of a single-chain peptide antagonist for the relaxin-3 receptor RXFP3. J. Am. Chem. Soc. 133, 4965–4974. 10.1021/ja110567j 21384867

[B12] Haugaard-Kedström L. M. Wong L. L. Bathgate R. A. Rosengren K. J. (2015). Synthesis and pharmacological characterization of a europium-labelled single-chain antagonist for binding studies of the relaxin-3 receptor RXFP3. Amino Acids 47, 1267–1271. 10.1007/s00726-015-1961-x 25792111

[B13] Hojo K. Hossain M. A. Tailhades J. Shabanpoor F. Wong L. L. Ong-Palsson E. E.. (2016). Development of a single-chain peptide agonist of the relaxin-3 receptor using hydrocarbon stapling. J. Med. Chem. 59, 16, 7445–7456. 10.1021/acs.jmedchem.6b00265 27464307

[B14] Hossain M. A. Haugaard-Kedström L. M. Rosengren K. J. Bathgate R. A. Wade J. D. (2015). Chemically synthesized dicarba H2 relaxin analogues retain strong RXFP1 receptor activity but show an unexpected loss of *in vitro* serum stability. Org. Biomol. Chem. 13, 10895–10903. 10.1039/C5OB01539A 26368576

[B15] Hossain M. A. Rosengren K. J. Haugaard-Jonsson L. M. Zhang S. Layfield S. Ferraro T.. (2008). The A-chain of human relaxin family peptides has distinct roles in the binding and activation of the different relaxin family peptide receptors. J. Biol. Chem. 283, 17287–17297. 10.1074/jbc.M801911200 18434306

[B16] Hu M. J. Shao X. X. Wang J. H. Wei D. Liu Y. L. Xu Z. G.. (2016). Identification of hydrophobic interactions between relaxin-3 and its receptor RXFP3: implication for a conformational change in the B-chain C-terminus during receptor binding. Amino Acids 48, 2227–2236. 10.1007/s00726-016-2260-x 27193232

[B17] Jayakody T. Marwari S. Lakshminarayanan R. Tan F. C. Johannes C. W. Dymock B. W.. (2016). Hydrocarbon stapled B chain analogues of relaxin-3 retain biological activity. Peptides 84, 44–57. 10.1016/j.peptides.2016.08.001 27498038

[B18] Ji Y. Majumder S. Millard M. Borra R. Bi T. Elnagar A. Y.. (2013). *In vivo* activation of the p53 tumor suppressor pathway by an engineered cyclotide. J. Am. Chem. Soc. 135, 11623–11633. 10.1021/ja405108p 23848581 PMC3767463

[B19] Keller R. L. J. (2004). The Computer Aided Resonance Assignment Tutorial. Goldau: Cantina Verlag.

[B20] Kuei C. Sutton S. Bonaventure P. Pudiak C. Shelton J. Zhu J.. (2007). R3(BDelta23 27)R/I5 chimeric peptide, a selective antagonist for GPCR135 and GPCR142 over relaxin receptor LGR7: *in vitro* and *in vivo* characterization. J. Biol. Chem. 282, 25425–25435. 10.1074/jbc.M701416200 17606621

[B21] Kumar J. R. Rajkumar R. Jayakody T. Marwari S. Hong J. M. Ma S.. (2017). Relaxin' the brain: a case for targeting the nucleus incertus network and relaxin-3/RXFP3 system in neuropsychiatric disorders. Br. J. Pharmacol. 174, 1061–1076. 10.1111/bph.13564 27597467 PMC5406295

[B22] Le-Nguyen D. Chiche L. Hoh F. Martin-Eauclaire M. F. Dumas C. Nishi Y.. (2007). Role of Asn(2) and Glu(7) residues in the oxidative folding and on the conformation of the N-terminal loop of apamin. Biopolymers 86, 447–462. 10.1002/bip.20755 17486576

[B23] Li C. Pazgier M. Liu M. Lu W. Y. Lu W. (2009). Apamin as a template for structure-based rational design of potent peptide activators of p53. Angew. Chem. Int. Ed. Engl. 48, 8712–8715. 10.1002/anie.200904550 19827079 PMC2845718

[B24] Liu C. Chen J. Kuei C. Sutton S. Nepomuceno D. Bonaventure P.. (2005). Relaxin-3/insulin-like peptide 5 chimeric peptide, a selective ligand for G protein-coupled receptor (GPCR)135 and GPCR142 over leucine-rich repeat-containing G protein-coupled receptor 7. Mol. Pharmacol. 67, 231–240. 10.1124/mol.104.006700 15465925

[B25] Liu C. Eriste E. Sutton S. Chen J. Roland B. Kuei C.. (2003). Identification of relaxin-3/INSL7 as an endogenous ligand for the orphan G-protein-coupled receptor GPCR135. J. Biol. Chem. 278, 50754–50764. 10.1074/jbc.M308995200 14522968

[B26] Ma S. Bonaventure P. Ferraro T. Shen P. J. Burazin T. C. Bathgate R. A.. (2007). Relaxin-3 in GABA projection neurons of nucleus incertus suggests widespread influence on forebrain circuits via G-protein-coupled receptor-135 in the rat. Neuroscience 144, 165–190. 10.1016/j.neuroscience.2006.08.072 17071007

[B27] Ma S. Olucha-Bordonau F. E. Hossain M. A. Lin F. Kuei C. Liu C.. (2009). Modulation of hippocampal theta oscillations and spatial memory by relaxin-3 neurons of the nucleus incertus. Learn. Mem. 16, 730–742. 10.1101/lm.1438109 19880588

[B28] Ma S. Smith C. M. Blasiak A. Gundlach A. L. (2017). Distribution, physiology and pharmacology of relaxin-3/RXFP3 systems in brain. Br. J. Pharmacol. 174, 1034–1048. 10.1111/bph.13659 27774604 PMC5406293

[B29] Mahalakshmi R. Balaram P. (2006). Non-protein amino acids in the design of secondary structure scaffolds. Methods Mol. Biol. 340, 71–94. 10.1385/1-59745-116-9:71 16957333

[B30] Marwari S. Poulsen A. Shih N. Lakshminarayanan R. Kini R. M. Johannes C. W.. (2019). Intranasal administration of a stapled relaxin-3 mimetic has anxiolytic- and antidepressant-like activity in rats. Br. J. Pharmacol. 176, 3899–3923. 10.1111/bph.14774 31220339 PMC6811745

[B31] Mccoll D. J. Honchell C. D. Frankel A. D. (1999). Structure-based design of an RNA-binding zinc finger. Proc. Natl. Acad. Sci. U.S.A. 96, 9521–9526. 10.1073/pnas.96.17.9521 10449725 PMC22241

[B32] Oller-Salvia B. Teixido M. Giralt E. (2013). From venoms to BBB shuttles: synthesis and blood-brain barrier transport assessment of apamin and a nontoxic analog. Biopolymers 100, 675–686. 10.1002/bip.22257 24281722

[B33] Patil N. A. Rosengren K. J. Separovic F. Wade J. D. Bathgate R. A. D. Hossain M. A. (2017). Relaxin family peptides: structure-activity relationship studies. Br. J. Pharmacol. 174, 950–961. 10.1111/bph.13684 27922185 PMC5406294

[B34] Phan T. Nguyen H. D. Goksel H. Mocklinghoff S. Brunsveld L. (2010). Phage display selection of miniprotein binders of the Estrogen Receptor. Chem. Commun. 46, 8207–8209. 10.1039/c0cc02727h 20871934

[B35] Rosengren K. J. Lin F. Bathgate R. A. Tregear G. W. Daly N. L. Wade J. D.. (2006). Solution structure and novel insights into the determinants of the receptor specificity of human relaxin-3. J. Biol. Chem. 281, 5845–5851. 10.1074/jbc.M511210200 16365033

[B36] Ryan P. J. Kastman H. E. Krstew E. V. Rosengren K. J. Hossain M. A. Churilov L.. (2013). Relaxin-3/RXFP3 system regulates alcohol-seeking. Proc. Natl. Acad. Sci. U.S.A. 110, 20789–20794. 10.1073/pnas.1317807110 24297931 PMC3870696

[B37] Shabanpoor F. Akhter Hossain M. Ryan P. J. Belgi A. Layfield S. Kocan M.. (2012). Minimization of human relaxin-3 leading to high-affinity analogues with increased selectivity for relaxin-family peptide 3 receptor (RXFP3) over RXFP1. J. Med. Chem. 55, 1671–1681. 10.1021/jm201505p 22257012

[B38] Shen Y. Bax A. (2013). Protein backbone and sidechain torsion angles predicted from NMR chemical shifts using artificial neural networks. J. Biomol. NMR 56, 227–241. 10.1007/s10858-013-9741-y 23728592 PMC3701756

[B39] Shepherd N. E. Hoang H. N. Abbenante G. Fairlie D. P. (2005). Single turn peptide alpha helices with exceptional stability in water. J. Am. Chem. Soc. 127, 2974–2983. 10.1021/ja0456003 15740134

[B40] Smith C. M. Chua B. E. Zhang C. Walker A. W. Haidar M. Hawkes D.. (2014). Central injection of relaxin-3 receptor (RXFP3) antagonist peptides reduces motivated food seeking and consumption in C57BL/6J mice. Behav. Brain Res. 268, 117–126. 10.1016/j.bbr.2014.03.037 24681162

[B41] Smith C. M. Shen P. J. Banerjee A. Bonaventure P. Ma S. Bathgate R. A.. (2010). Distribution of relaxin-3 and RXFP3 within arousal, stress, affective, and cognitive circuits of mouse brain. J. Comp. Neurol. 518, 4016–4045. 10.1002/cne.22442 20737598

[B42] Swedberg J. E. Schroeder C. I. Mitchell J. M. Fairlie D. P. Edmonds D. J. Griffith D. A.. (2016). Truncated glucagon-like peptide-1 and exendin-4 alpha-conotoxin pl14a peptide chimeras maintain potency and alpha-helicity and reveal interactions vital for cAMP signaling *in vitro*. J. Biol. Chem. 291, 15778–15787. 10.1074/jbc.M116.724542 27226591 PMC4957059

[B43] Tanaka M. Iijima N. Miyamoto Y. Fukusumi S. Itoh Y. Ozawa H.. (2005). Neurons expressing relaxin 3/INSL 7 in the nucleus incertus respond to stress. Eur. J. Neurosci. 21, 1659–1670. 10.1111/j.1460-9568.2005.03980.x 15845093

[B44] Volkman B. F. Wemmer D. E. (1997). Deletion of a single amino acid changes the folding of an apamin hybrid sequence peptide to that of endothelin. Biopolymers 41, 451–460. 9080780 10.1002/(SICI)1097-0282(19970405)41:4<451::AID-BIP9>3.0.CO;2-L

[B45] Wang C. K. Craik D. J. (2018). Designing macrocyclic disulfide-rich peptides for biotechnological applications. Nat. Chem. Biol. 14, 417–427. 10.1038/s41589-018-0039-y 29662187

[B46] Wang C. K. Gruber C. W. Cemazar M. Siatskas C. Tagore P. Payne N.. (2014). Molecular grafting onto a stable framework yields novel cyclic peptides for the treatment of multiple sclerosis. ACS Chem. Biol. 9, 156–163. 10.1021/cb400548s 24147816 PMC3898541

[B47] Weston C. J. Cureton C. H. Calvert M. J. Smart O. S. Allemann R. K. (2004). A stable miniature protein with oxaloacetate decarboxylase activity. Chembiochem 5, 1075–1080. 10.1002/cbic.200300805 15300830

[B48] Wishart D. S. Bigam C. G. Holm A. Hodges R. S. Sykes B. D. (1995). 1H, 13C and 15N random coil NMR chemical shifts of the common amino acids. I. Investigations of nearest-neighbor effects. J. Biomol. NMR 5, 67–81. 10.1007/BF00227471 7881273

[B49] Wong L. L. L. Scott D. J. Hossain M. A. Kaas Q. Rosengren K. J. Bathgate R.. (2018). Distinct but overlapping binding sites of agonist and antagonist at the relaxin family peptide 3 (RXFP3) receptor. J. Biol. Chem. 293, 15777–15789. 10.1074/jbc.RA118.002645 30131340 PMC6187618

[B50] Wu J. Jiang H. Bi Q. Y. Luo Q. S. Li J. J. Zhang Y.. (2014). Apamin-mediated actively targeted drug delivery for treatment of spinal cord injury: more than just a concept. Mol. Pharm. 11, 3210–3222. 10.1021/mp500393m 25098949

[B51] Wüthrich K. (1986). NMR of Proteins and Nucleic Acids. New York, NY: John Wiley and Sons.

[B52] Zhang C. Chua B. E. Yang A. Shabanpoor F. Hossain M. A. Wade J. D. (2015a). Central relaxin-3 receptor (RXFP3) activation reduces elevated, but not basal, anxiety-like behaviour in C57BL/6J mice. Behav. Brain Res. 292, 125–132. 10.1016/j.bbr.2015.06.010 26057358

[B53] Zhang Y. Deng C. Liu S. Wu J. Chen Z. Li C.. (2015b). Active targeting of tumors through conformational epitope imprinting. Angew. Chem. Int. Ed. Engl. 54, 5157–5160. 10.1002/anie.201412114 25727886

