# Supplementary material for: Development of Relaxin-3 Agonists and Antagonists Based on Grafted Disulfide-Stabilized Scaffolds
Source: Front Chem. 2020 Feb 18;8:87. doi: 10.3389/fchem.2020.00087 (PMC7039932; doi:10.3389/fchem.2020.00087)
Supplement: Supplementary file 1 [file Table_1.DOCX]

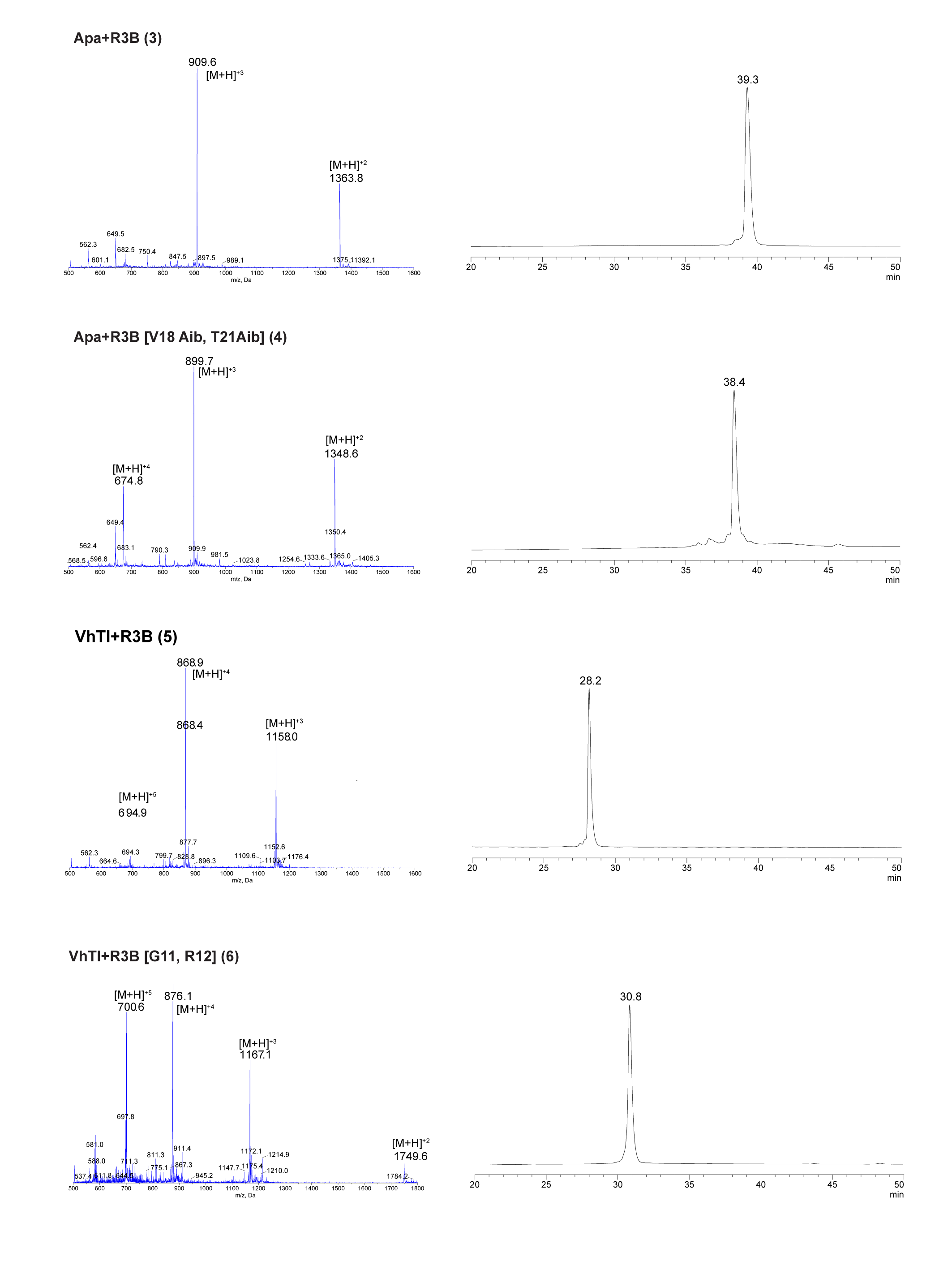


**Figure S1.** MS spectra and corresponding analytical HPLC traces of oxidised and purified analogues **3** – **6**.


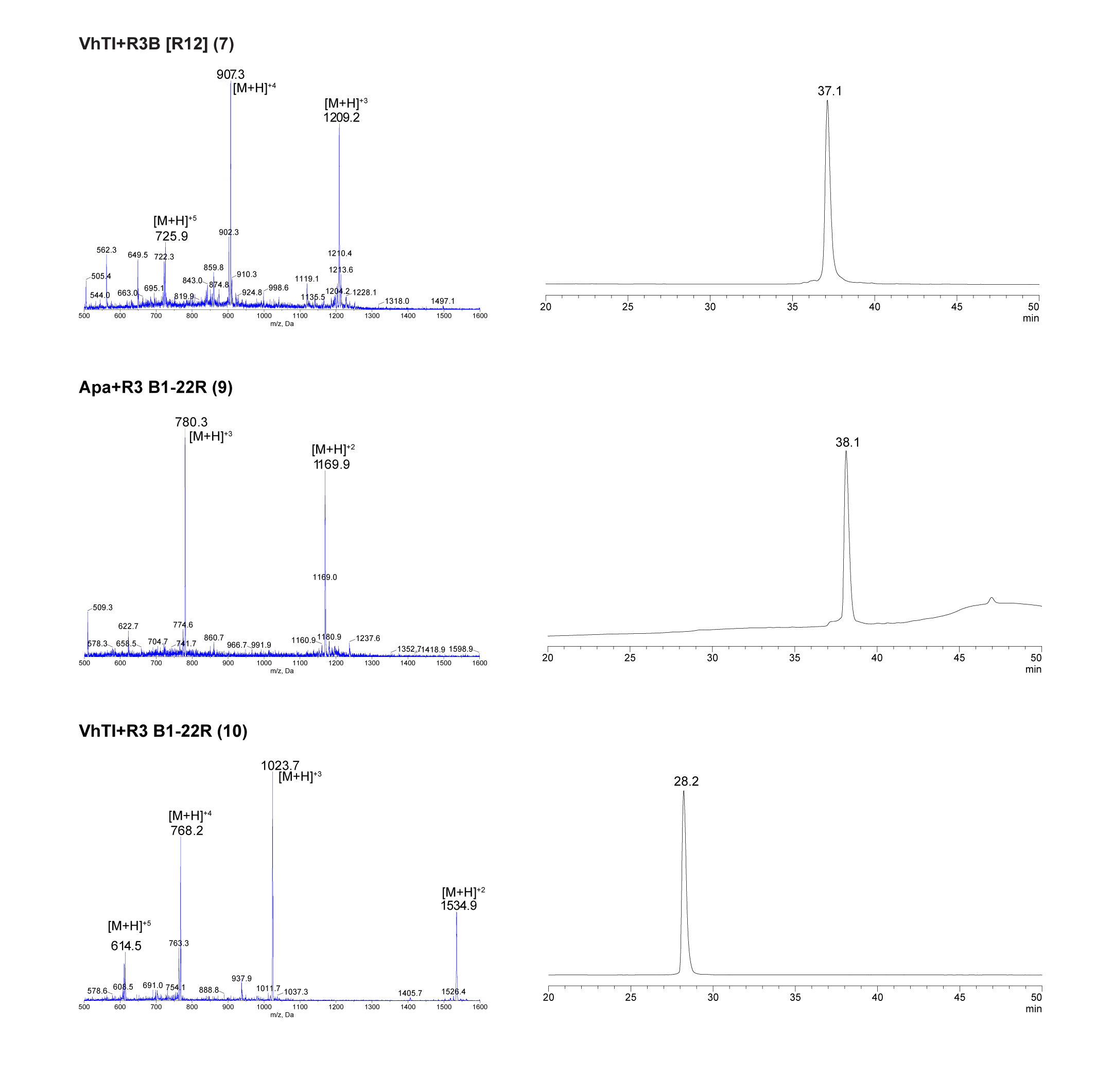
**Figure S2.** MS spectra and the corresponding analytical HPLC trace of purified final product if analogues **7, 9** and **10**.


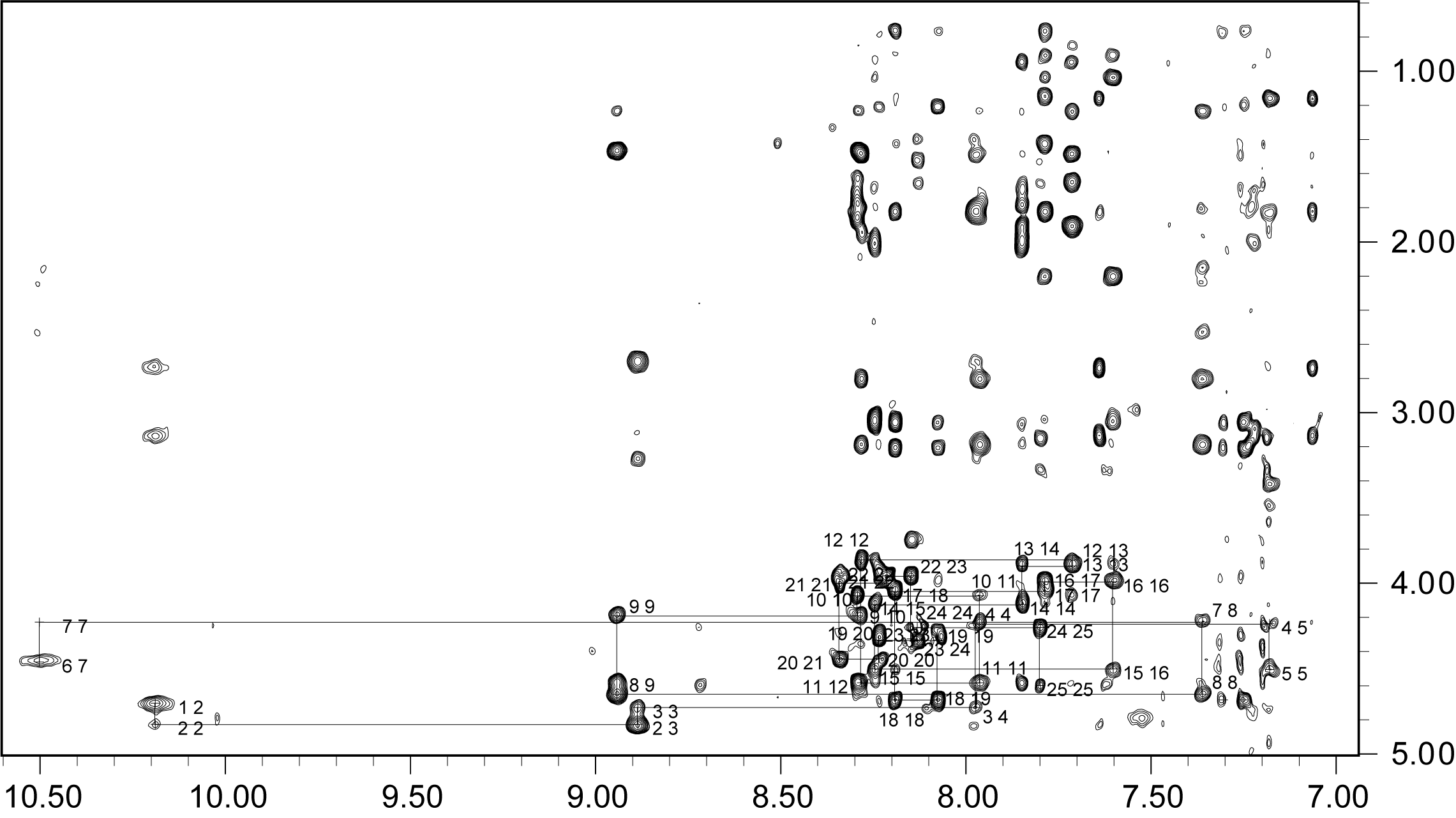


**Figure S3**. NOESY spectrum recorded at 298K with a mixing time of 200 ms of analogue **3.** The sequential walk highlighting the NMR spin system assignments are shown by connecting lines. Hα-HN cross peaks are labelled with residue numbers.


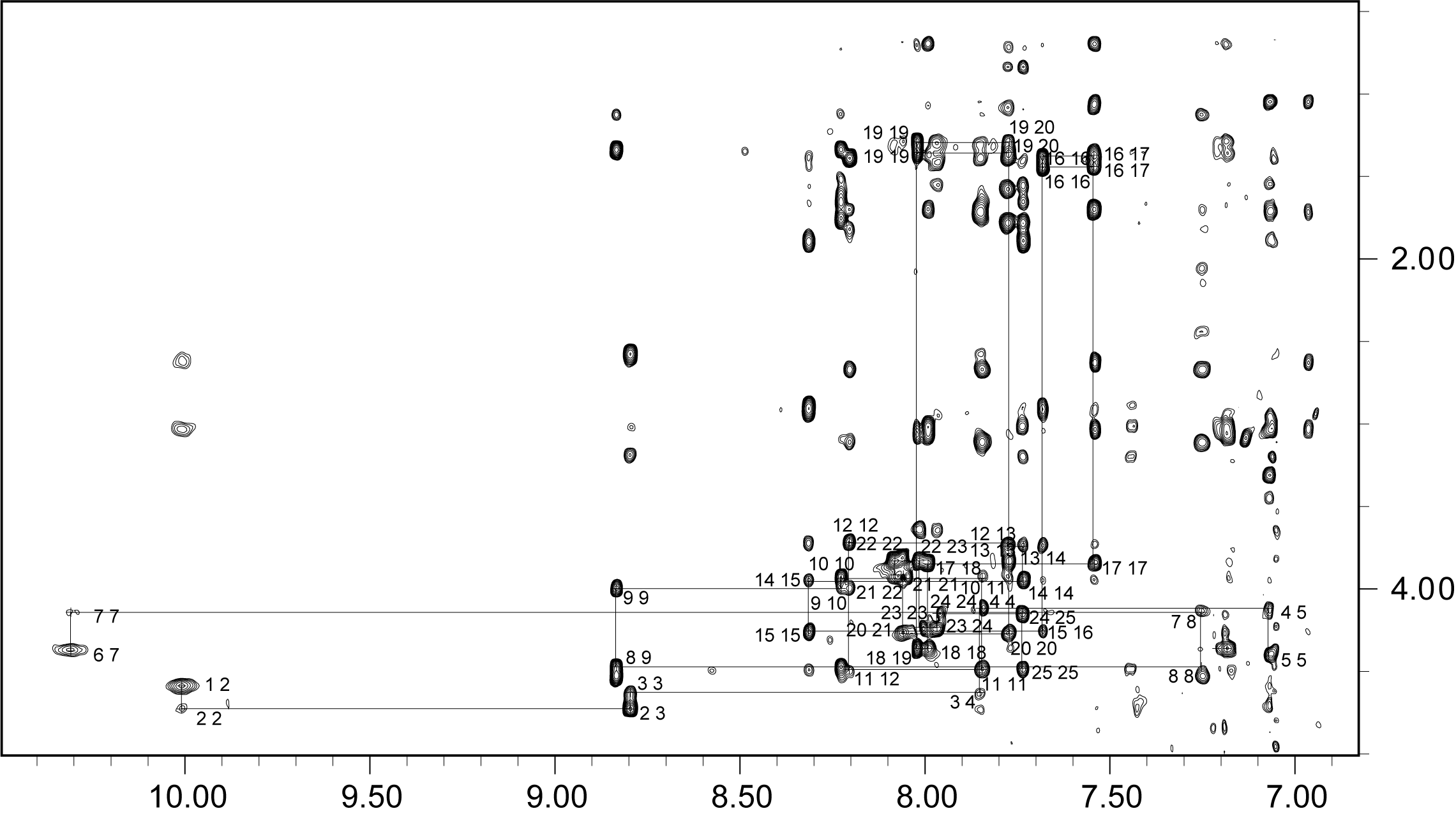


**Figure S4**. NOESY spectrum recorded at 298K with a mixing time of 200 ms of analogue **4.** The sequential walk highlighting the NMR spin system assignments are shown by connecting lines. Hα-HN cross peaks are labelled with residue numbers.


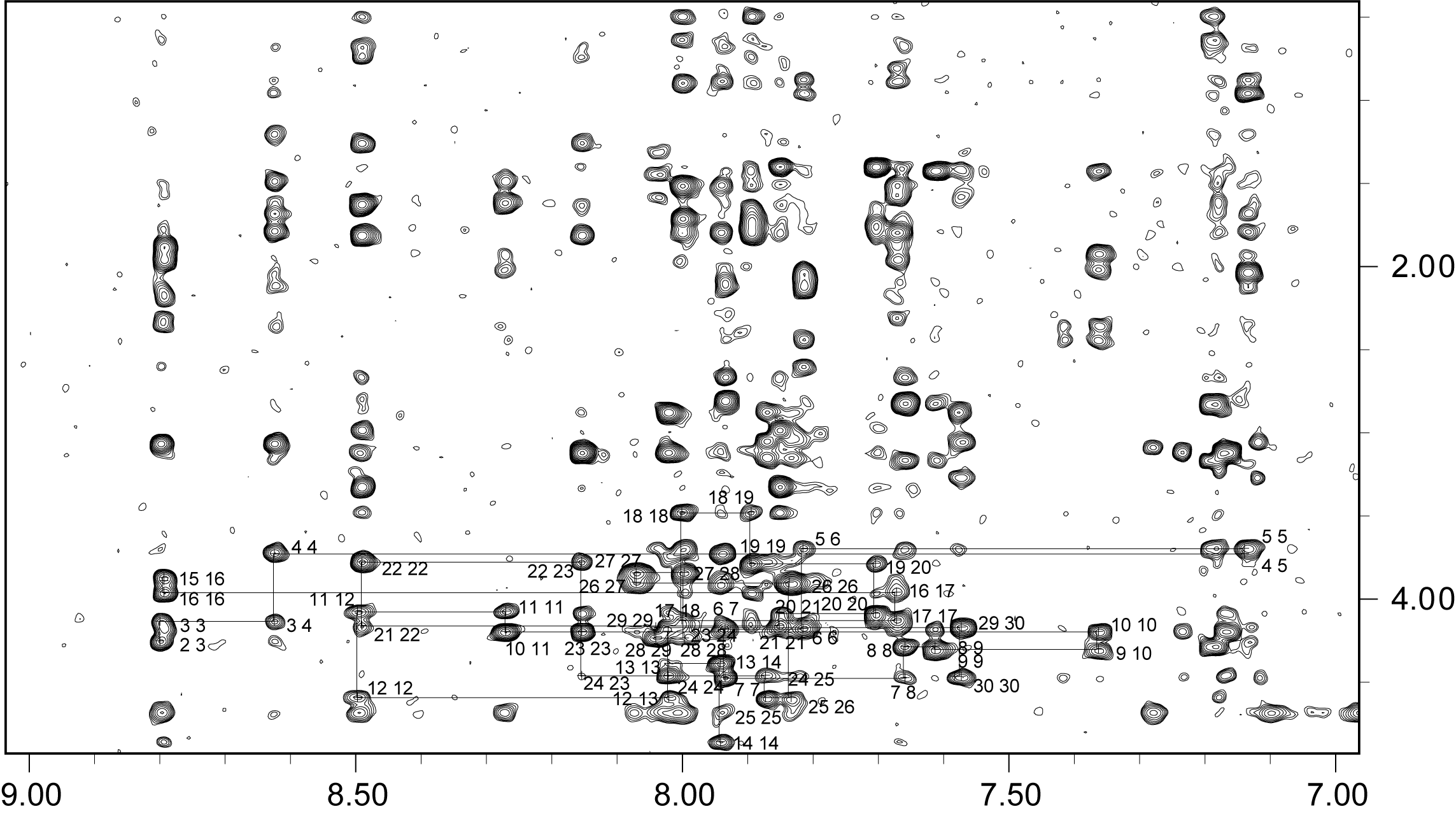


**Figure S5**. NOESY spectrum recorded at 298K with a mixing time of 200 ms of analogue **5.** The sequential walk highlighting the NMR spin system assignments are shown by connecting lines. Hα-HN cross peaks are labelled with residue numbers.


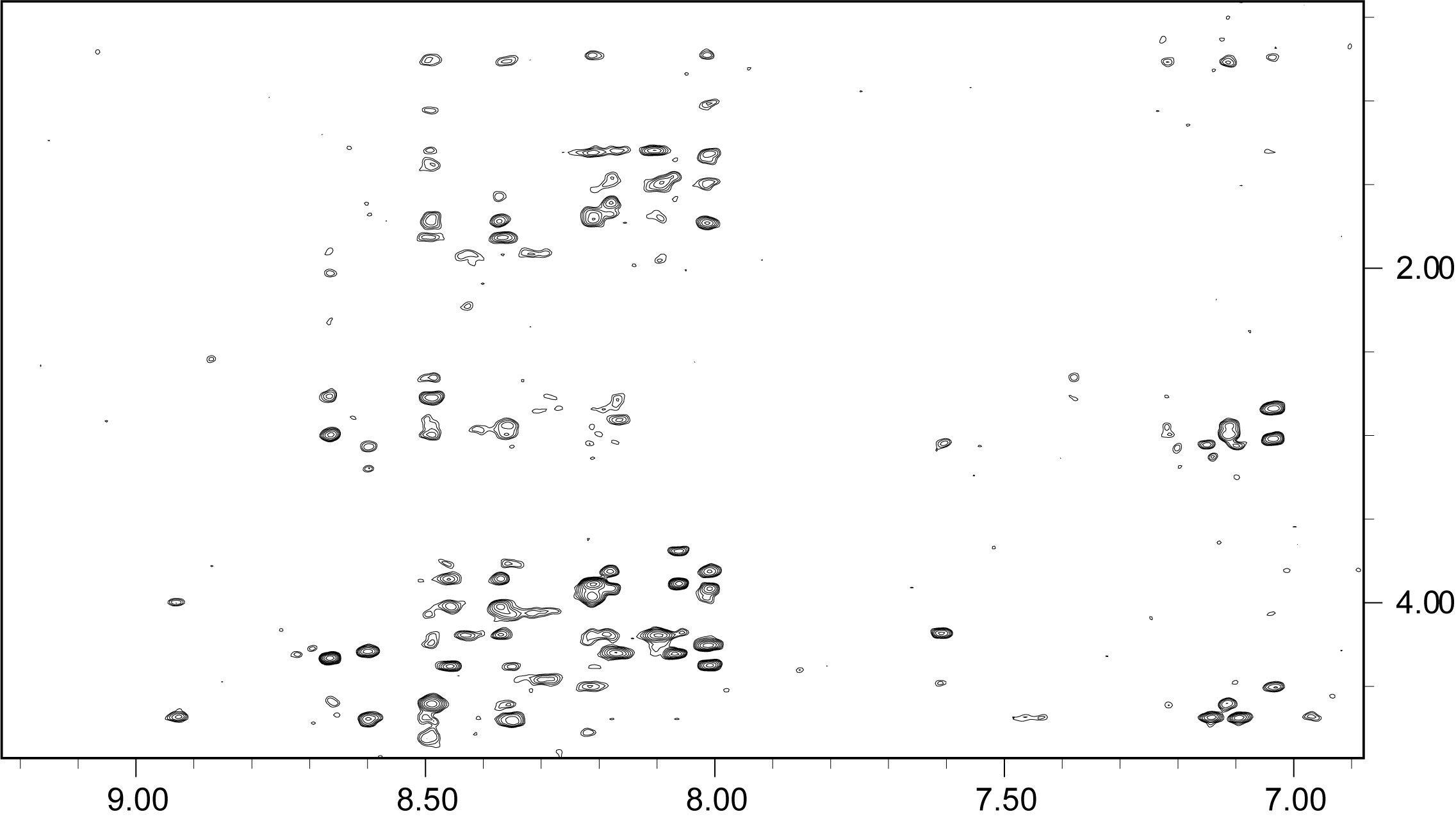


**Figure S6**. NOESY spectrum recorded at 298K with a mixing time of 200 ms of analogue **6.** Poor dispersion and lack of NOEs are consistent with a lack or ordered structure.


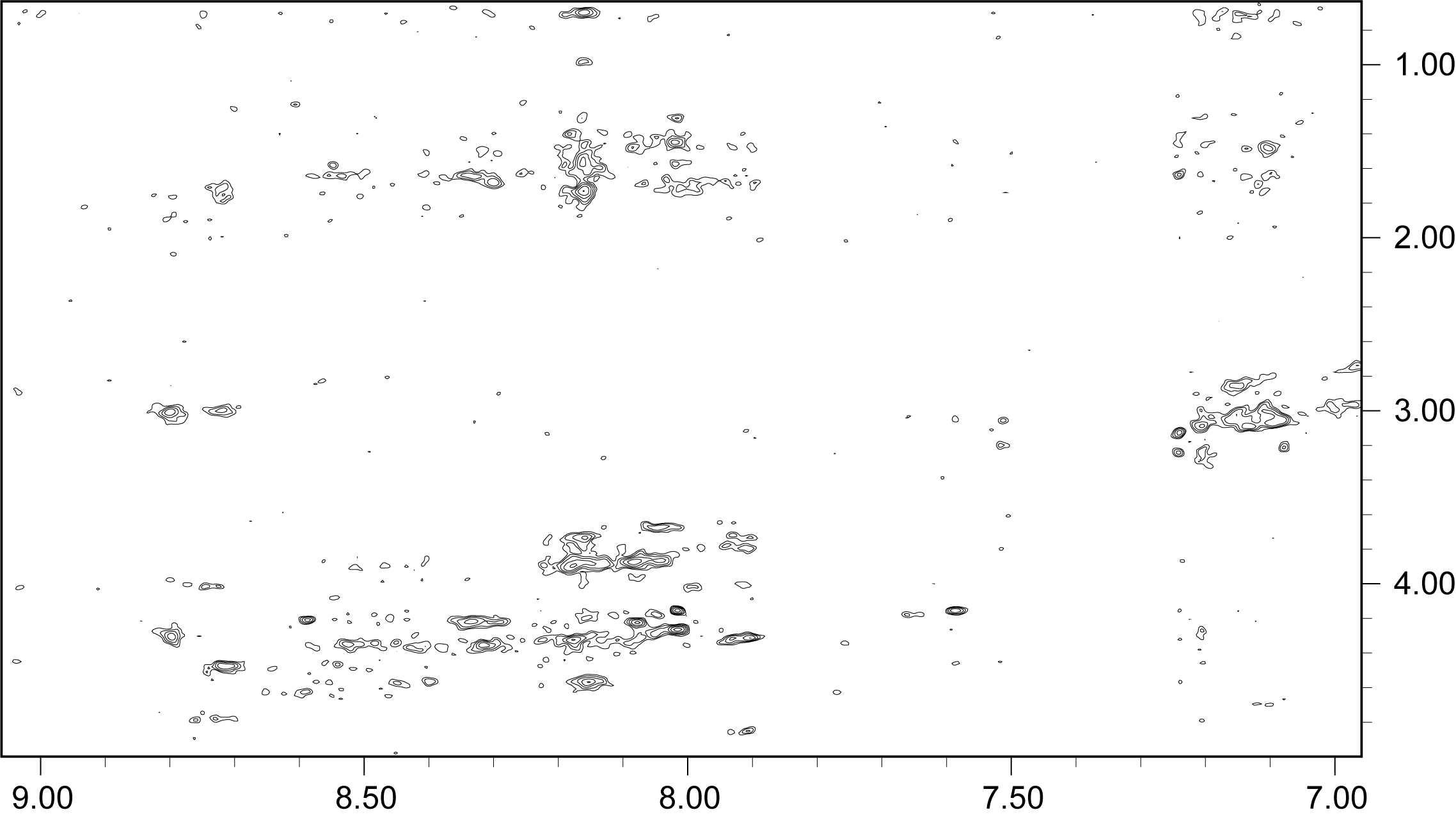


**Figure S7**. NOESY spectrum recorded at 298K with a mixing time of 200 ms of analogue **7.** Poor dispersion, lack of NOEs and severe line broadening are consistent with a lack of ordered structure and potential aggregation.


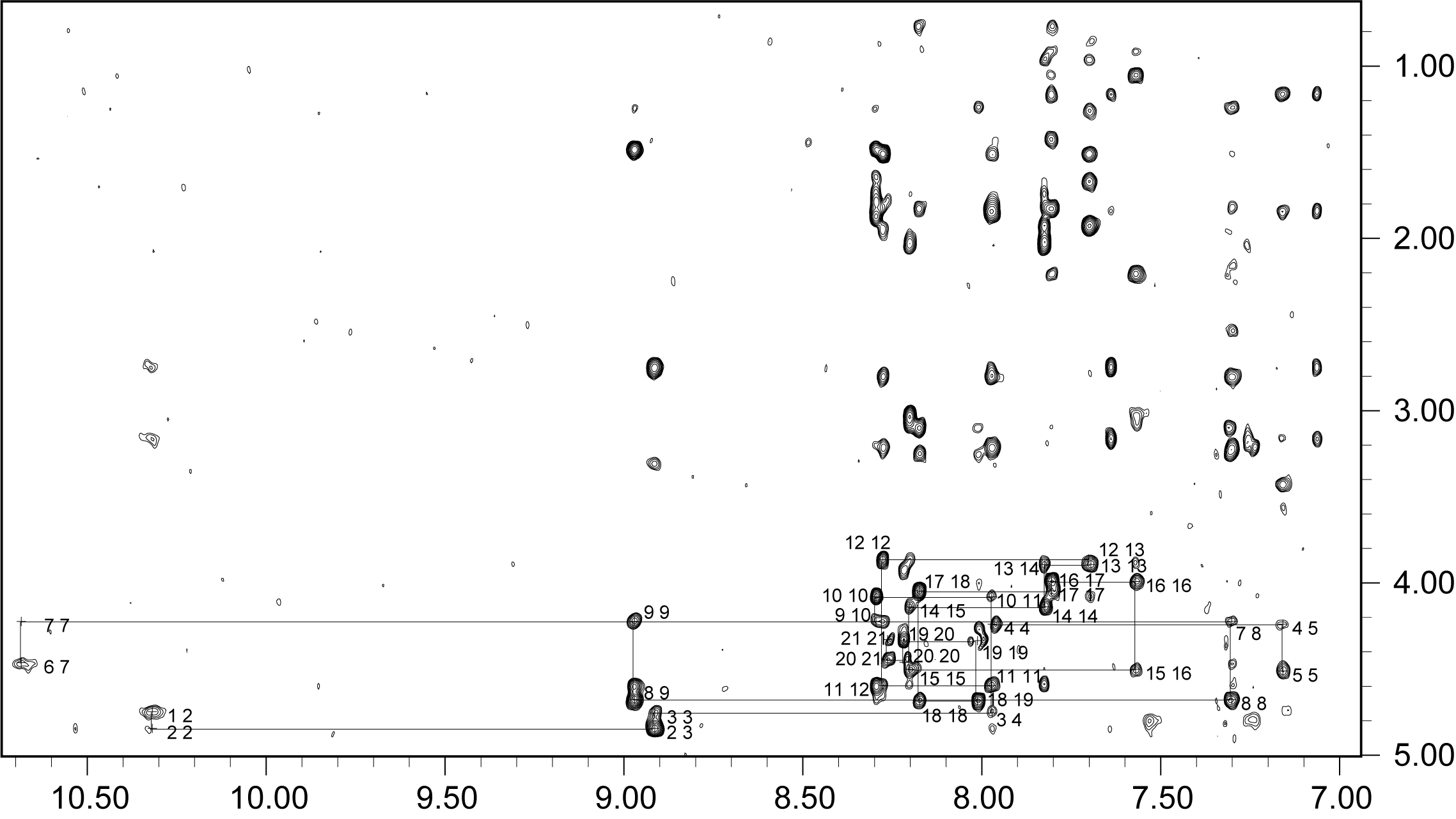


**Figure S8**. NOESY spectrum recorded at 298K with a mixing time of 200 ms of analogue **9.** The sequential walk highlighting the NMR spin system assignments are shown by connecting lines. Hα-HN cross peaks are labelled with residue numbers.


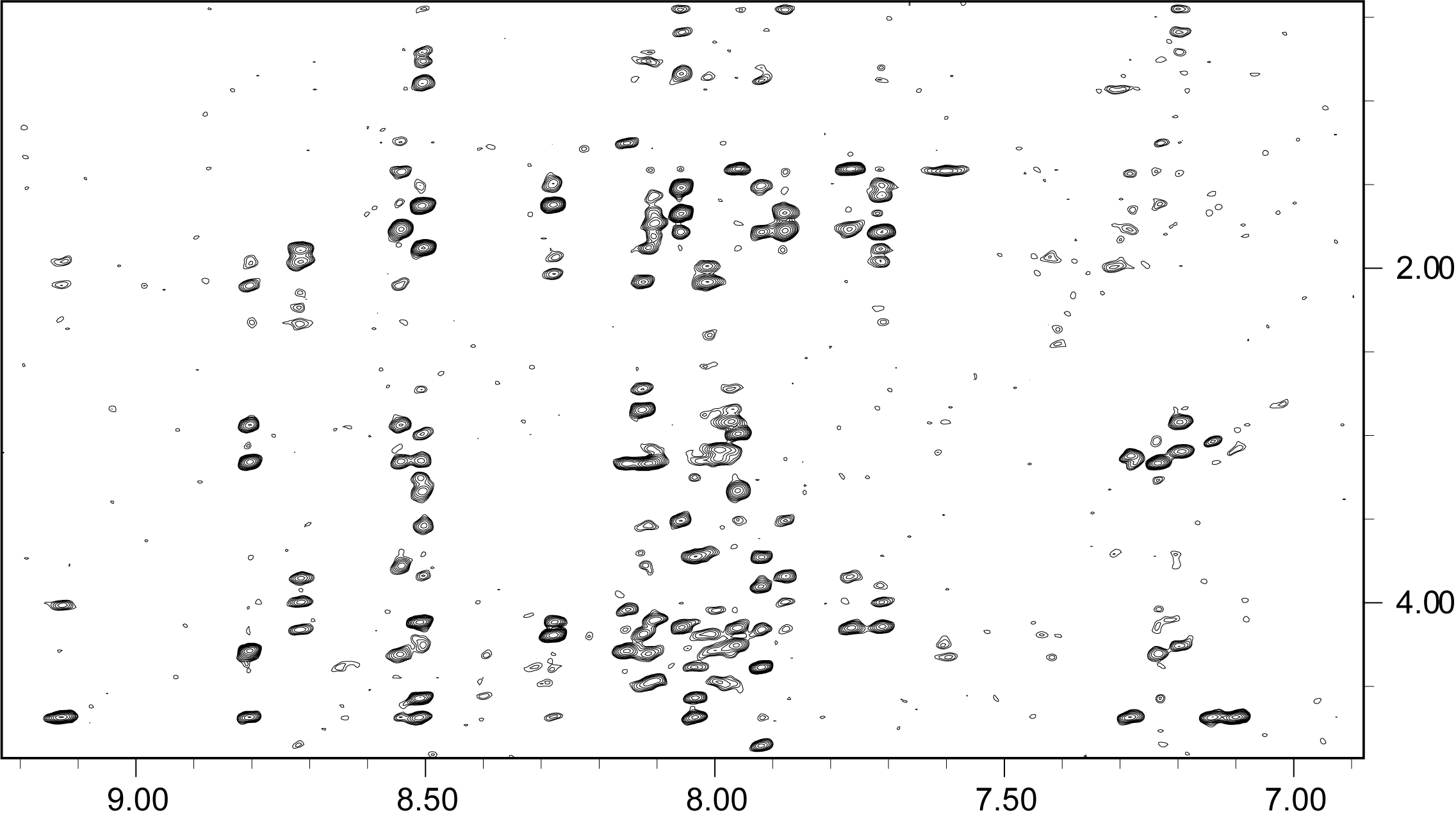


**Figure S9**. NOESY spectrum recorded at 298K with a mixing time of 200 ms of analogue **10.** The spectrum is similar to that of analogue **5**, which highlights that both the agonist and antagonist variants grafted on VhTI adopt similar well-structured folds.


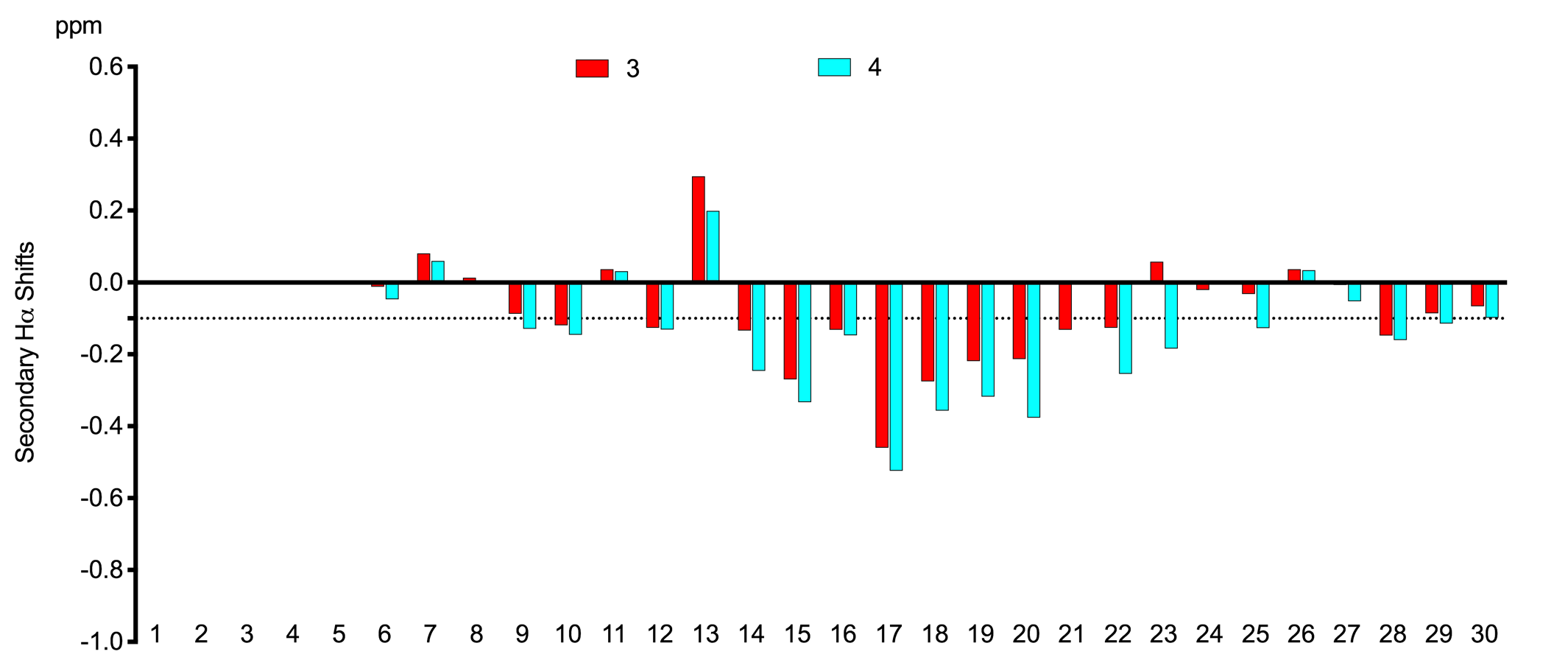


**Figure S10.** Comparison of secondary Hα chemical shifts for analogue **3** and analogue **4**. The stretch of negative values from 15-24 correspond to the relaxin-3 B-chain helix. The more negative values in the later part of this helix show that the helical nature is increased by the incorporation of Aib residues. Residues numbers on the X-axis relate to the longest peptide sequence in the main manuscript, analogue **5**.

**Table S1. Structural statistics from NMR based structure calculations**

|  | **Analogue 3** | **Analogue 9** | **Analogue 5** |
| --- | --- | --- | --- |
| **MolProbity statistics** |  |  |  |
| Clashes (> 0.4 Å/1000 atoms) | 6.73 ± 3.61 | 5.40 ± 4.13 | 3.81 ± 2.54 |
| Poor rotamers | 0.25 ± 0.64 | 0.25 ± 0.55 | 1.45 ± 1.19 |
| Ramachandran outliers (%) | 1.43 ± 2.93 | 0.50 ± 2.24 | 0.31 ± 1.40 |
| Ramachandran favoured (%) | 82.14 ± 8.82 | 94.00 ± 6.81 | 90.00 ± 5.88 |
| MolProbity score | 2.10 ± 0.20 | 1.58 ± 0.22 | 2.08 ± 0.28 |
| MolProbity score percentile | 69.80 ± 10.53 | 91.60 ± 5.93 | 69.80 ± 14.05 |
| **Distance restraints** |  |  |  |
| Intraresidue (i-j = 0) | 111 | 69 | 117 |
| Sequential (/i-j/ = 1) | 85 | 54 | 106 |
| Medium range (/i-j/ ≤ 5) | 45 | 13 | 88 |
| Long range (/i-j/ > 5) | 11 | 6 | 27 |
| Hydrogen bonds | 6 | 7 | NA |
| Total | 258 | 149 | 338 |
| **Dihedral angle restraints** |  |  |  |
| φ (phi) | 11 | 12 | NA |
| ψ (psi) | 12 | 13 | NA |
| Total | 23 | 25 | NA |
| Average pairwise r.m.s.d.*^a^* (Å) |  |  |  |
| Backbone atoms | 0.14 ± 0.07 | 0.80 ± 0.34 | 1.23 ± 0.40*^b^* |
| Heavy atoms | 1.18 ± 0.25 | 1.86 ± 0.40 | 2.37 ± 0.53 |

*a* Calculated from 20 refined structures over residues 1-15

*b* Calculated from 20 refined structures over residues 3-25
